# Levatorplasty’s role in rectal prolapse management for patients with wide pelvic hiatus: a cohort study

**DOI:** 10.1186/s12893-024-02693-9

**Published:** 2025-01-11

**Authors:** Mohamed Yehia Elbarmelgi, Ali Ahmed Shafik, Shady Fathy Badee, Osama Refaie Mohamed, Mohamed Tamer

**Affiliations:** Cairo university, Giza, Egypt

**Keywords:** Rectal prolapse, Pelvic hiatus, Recurrence, Defecography

## Abstract

**Background and aims:**

Rectal prolapse (RP) is a debilitating condition and can cause symptoms of fecal incontinence, obstructed defecation, incomplete evacuation of the rectum, and pain. In recent years, there has been increasing evidence that prolapse development is strongly associated with pelvic hiatus (GH) size (normal 4.5 +/- 0.7). Multiple surgical studies have suggested that an enlarged pelvic hiatus may be associated with prolapse recurrence. The main aim of this study is to assess the role of combining levatorplasty -with different rectal prolapse surgeries- on recurrence rate and improvement of symptoms in patients that were treated for rectal prolapse with wide pelvic hiatus.

**Patients and methods:**

Our study is a combined retrospective and prospective cohort study which included sixty patients with rectal prolapse with wide pelvic hiatus, they were divided into two groups (each group thirty patients). One group underwent rectal prolapse repair, the other group underwent rectal prolapse repair with levatorplasty.

**Results:**

Levatorplasty group showed improvement in Wexner score, recurrence rate in comparison to the other group. Both groups showed similar results in postoperative pain and dyspareunia.

**Conclusion:**

Rectal prolapse repair combined with levatorplasty in patients complaining of rectal prolapse with wide pelvic hiatus have better outcome mainly in decreasing recurrence rate.

## Introduction

Rectal prolapse and internal intussusception is a range of anatomical abnormalities involving descent of rectal wall -either partially or completely- that may be associated with either incontinence or obstructed defecation due to pelvic floor dysfunction [1]. Although those are benign conditions, they may cause a lot of symptoms that can be very debilitating. A lot of factors may lead to the development of rectal prolapse such as widening the levator hiatus, a patulous anal sphincter, and loss of the rectal sacral attachments. Previously just restoring normal anatomy was regarded as success. Yet, the need for many processes to address this issue shows that obtaining satisfactory results can be challenging [2].

Rectal prolapse is most frequently seen in women above the age of 70 – although many exceptions have been reported recently- and has been linked to a variety of pelvic floor problems including vaginal prolapse, enterocele, cystocele, rectocele, and urine incontinence. Generally, multiparity and weak pelvic floor are the main factors that may lead to the development of rectal prolapse or intussusception. Rectal prolapse is six times more common in women than in men [3].

Surgery is the mainstay of rectal prolapse treatment. However, there are a wide range of used techniques being reported in the literature which can be performed either through abdominal or perineal approaches. The patient’s comorbidity, age, the main presentation (incontinence, constipation or just protrusion of tissues) and the surgeon’s preferences all influence the surgical strategy [4].

The puborectalis (PR) muscle is one of the three muscular slings of the levator ani (LA) muscle, which creates the pelvic floor diaphragm. The PR muscle is formed by the most medial fibers of the pubococcygeus, which travel around the rectum at the anorectal junction and are condensed into the anterior portion of the obturator internus fascia [5, 6].

Anal sphincters, the perineal body, and the rectoceles can all be harmed by repeated pregnancy, delivery and perineal trauma [7]. The levator hiatus widens after delivery, starting from 13.8 ± 1.7 cm2 in nulliparous woman to 24.2 ± 2.1 cm2 after delivery [8]. The initial birth caused either a complete or incomplete LA muscle injury, which is the main cause of this anatomical alteration [9].

## What does this study add to literature?

As discussed above, widening of the Levator hiatus may play an important role in the development of rectal prolapse. The role of repair of the levator hiatus during surgery for rectal prolapse are not much discussed in the available literature. In this study we are investigating the role of combining Levatorplasty to different surgical repairs of rectal prolapse in decreasing the recurrence rate and control of symptoms.

## Patients and methods

### Study type

This study is a combined retrospective and prospective Cohort study studying the role of levatorplasty in management of rectal prolapse in patients with wide pelvic hiatus.

### Objectives

The **Primary objective** of this study was to assess the role of adding levatorplasty on recurrence rate and improvement of symptoms in patients that was treated from rectal prolapse with wide pelvic hiatus. The **Secondary Objectives** was to assess the effect of adding levatorplasty on post-operative pain, post-operative time, post-operative hospital stays and urogenital complications as dyspareunia.

### Study settings

The study was held over a period of 20 months in Cairo university hospitals during the period from July 2021 till February 2023 on patients presenting with symptomatizing rectal prolapse with wide pelvic hiatus.

### Patients’ population

Patients presenting with rectal prolapse with wide pelvic hiatus candidate for rectal prolapse surgery were divided into two groups: one group underwent rectal prolapse surgery with Levatorplasty and this group was the new series of patients, the other group was retrieved from our records and included patients complaining of symptomatizing rectal prolapse also with wide pelvic hiatus but underwent only rectal prolapse surgery without levatorplasty (control group). Surgery addressing the rectal prolapse only without levatorplasty (control group) was the standard of our practice until the idea of repairing the pelvic hiatus evolved (levatorplasty group) and we select patients that are suitable for the study from our records as control group. This explains the combined retrospective and prospective nature of the study. We also compared selected patients of both groups regarding several aspects as discussed below to decrease biases as much as possible.

### Demographic data

Comparison between both groups regarding demographic data are represented in Table (1). There was no statistical significance between both groups regarding age, gender, BMI (body mass index) and ASA grade.

**Table 1:** Comparison between both groups regarding demographic data

		With levatorplasty	Without levatorplasty	Total (*N* = 60)	*P*-value	Sig.
		No. = 30	No. = 30			
**Age**	Mean	47.47 ± 4.59	47.13 ± 6.00	47.30 ± 5.30 30–58	0.235	NS
	Range	30–58	32–56			
**Gender**	Females	28 (93.3%)	28 (93.3%)	56 (93.3%) 4 (6.7%)	1	NS
	Males	2 (6.7%).	2 (6.7%).			
**BMI**	Mean Range	36.73 ± 2.26 33 to 48Kg	37.67 ± 3.47 33 to 48Kg	37.20 ± 2.94 33 to 48Kg	0.222	NS
**ASA grade**		ASA1 (14 patients), ASA 2 (15 patients) and ASA 3 (1 patient)	ASA 1 (13 patients), ASA 2 (14 patients), ASA 3 (3 patients)		1	NS

### Inclusion criteria

The subjects were considered appropriate candidates for the study if they were: Willing to give consent and comply with the evaluation and treatment schedule, patients show Rectal prolapse with wide pelvic hiatus **{normal dimensions 4.5 +/- 0.7Cm} selected by MRI defecography** [8, 9] from both sex& all age groups, Patients are generally fit for anesthesia and surgery.

### Exclusion criteria

Exclusion criteria to this study includes:

1-Mental incompetence.

2- Inability or unwillingness of the patient to change their lifestyle post- operatively.

3- Drug, alcohol, or other substance addiction.

4- Psychological instability.

5- Uncontrolled diabetes mellitus (DM).

6- Connective Tissue Diseases.

7- Recurrent rectal prolapse.

8- Previous rectal surgery.

9- Neurological deficit Patients.

10- Paraplegic Patients.

11- Rectal prolapse occurred after Spine surgery or trauma.

12- Previous Levator ani tear (trauma or surgery).

**Fig. 1:**
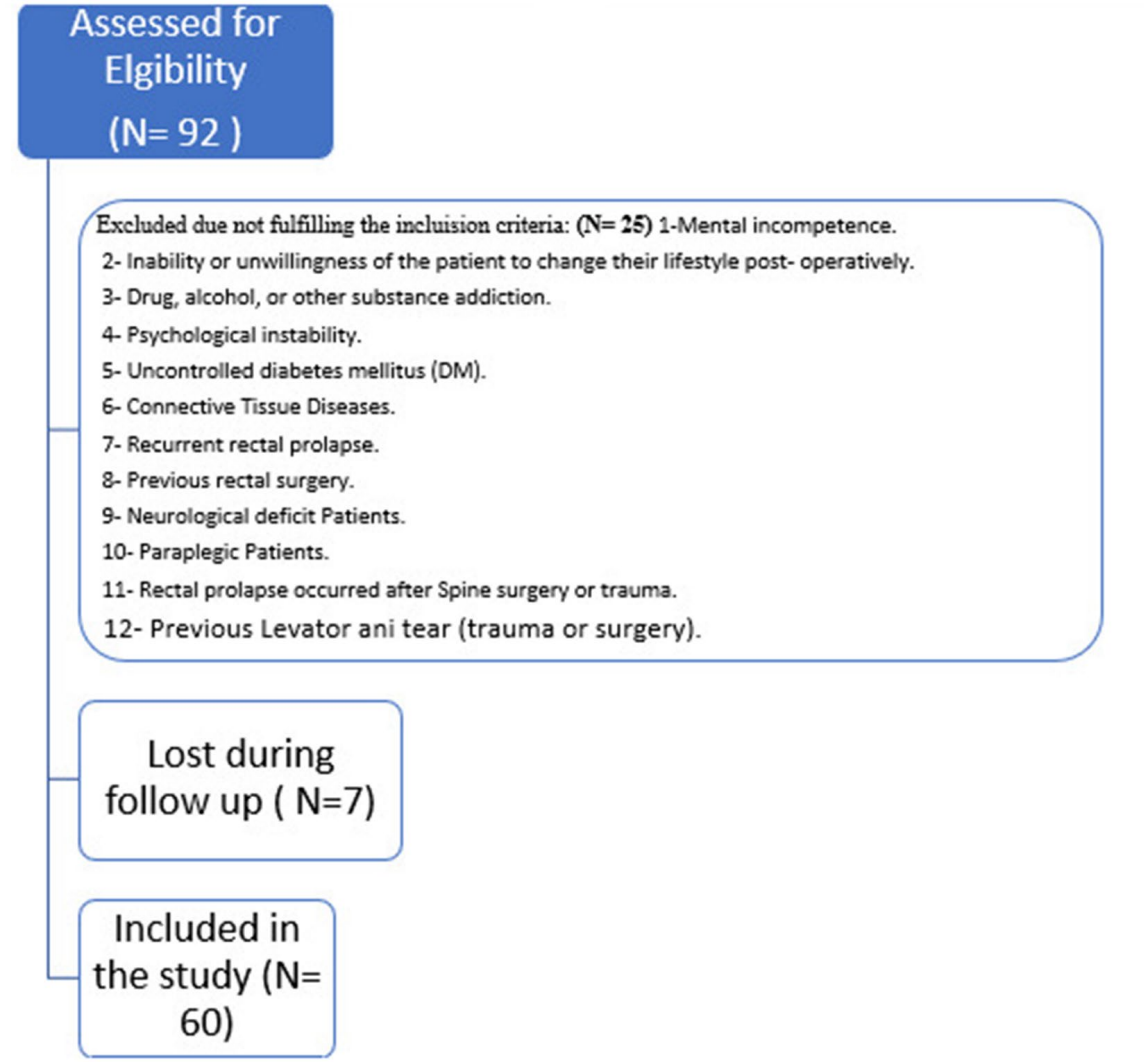
Flow chart of included and excluded patient

Look at the flow chart at Fig. (1) for exclusion criteria and enrollment of patients.

### Interventions

All patients underwent Rectal prolapse surgery either Altemeier’s procedure, Stappled Trasns Anal Rectal Resection (STARR procedure), Ventral Mesh Rectopexy (VMR) or pelvic organ prolapse surgery (POPS) as a primary one-stage procedure.

Thirty patients were conducted to rectal prolapse repair - by one of the above procedures according to the patient condition- without levatorplasty & thirty patients conducted to rectal prolapse repair with levatorplasty.

Preoperative evaluation followed the same standard protocol and included a thorough history, complete laboratory workup, psychological counseling, counseling by a dietician and Wexner score [10]. MRI Defecography was done to all patients to assess rectal prolapse, associated urogenital prolapse, and pelvic floor (Levator) hiatus. (Fig. 4)

Before surgery the following data was obtained: age, weight, body mass index (BMI), duration of disease, current treatment, presence of constipation or incontinence, Wexner score, medical history, history of any previous surgery and dyspareunia.

### Operative technique

#### Surgical procedure

Standard Rectal prolapse surgery was done either ALTEMEIER’S or STARR or Ventral Mesh Rectopexy or POPS according to the patient condition, for example if there was a multiple pelvic organ prolapse POPS procedure were planned. Another example if the patient is vulnerable, a perineal procedure (either Altemeier or STARR) was planned.

**Fig. 2:**
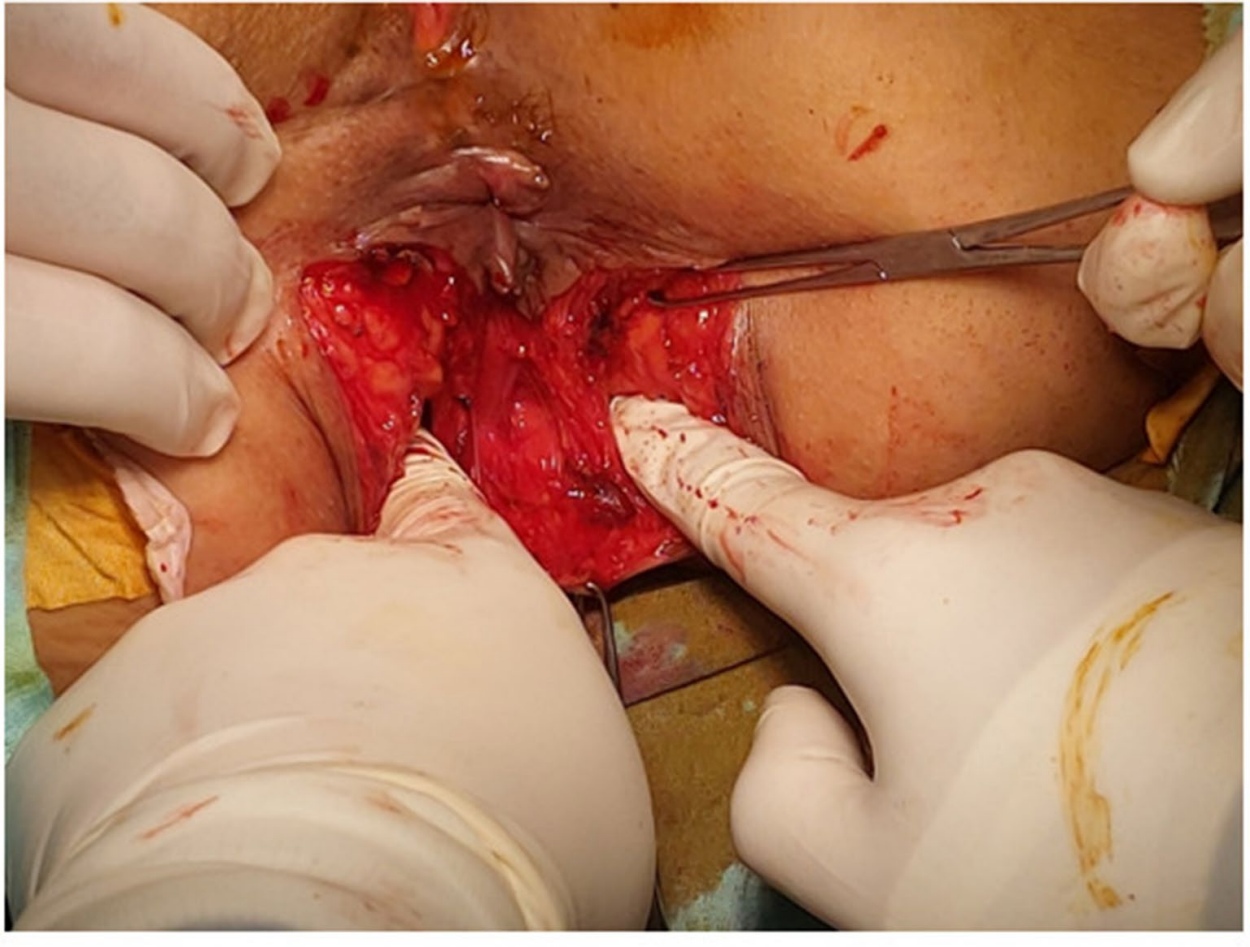
Levatorplasty (intraoperative showing wide pelvic hiatus before correction)

**Fig. 3:**
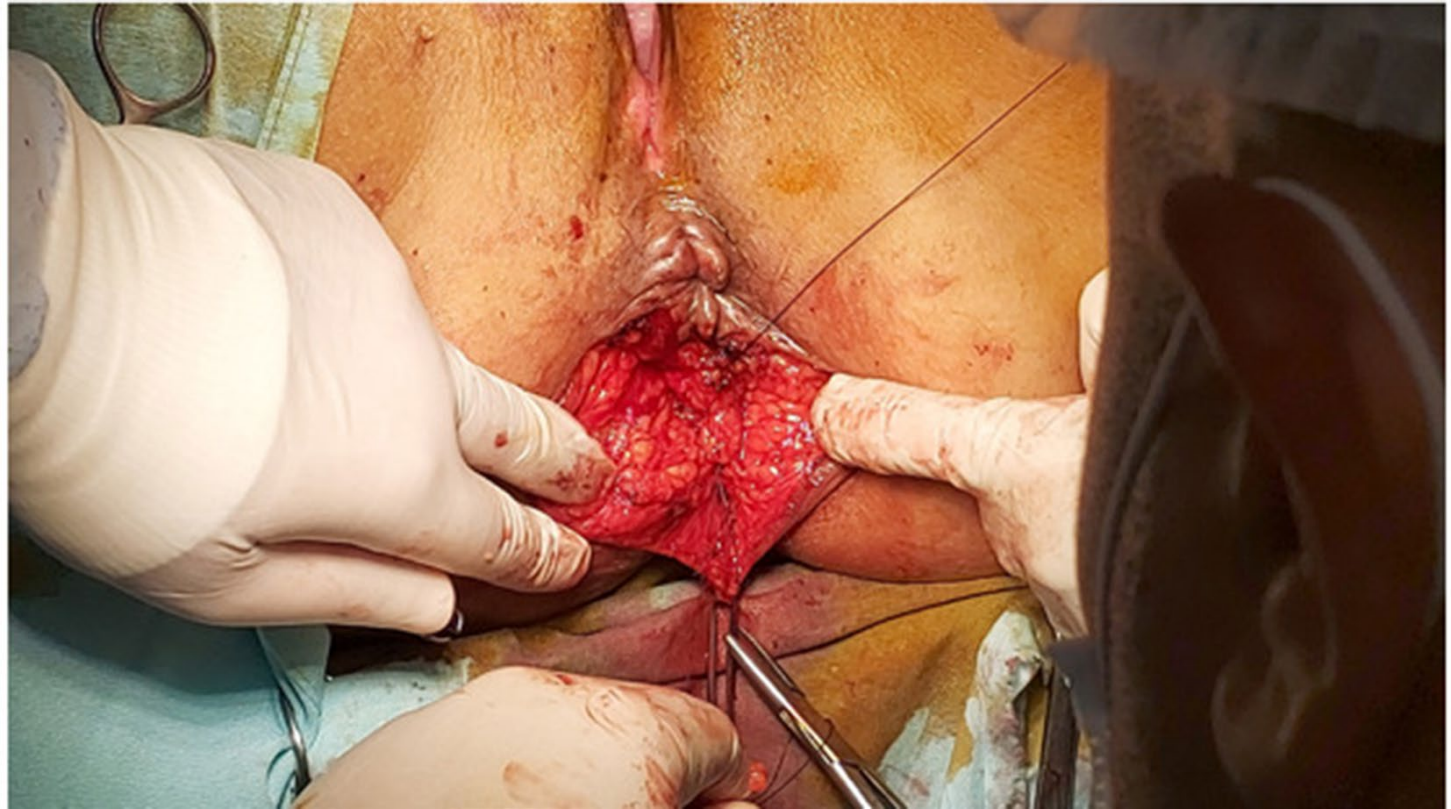
Levatorplasty (intraoperative) showing corrected pelvic hiatus after surgery

**Fig. 4:**
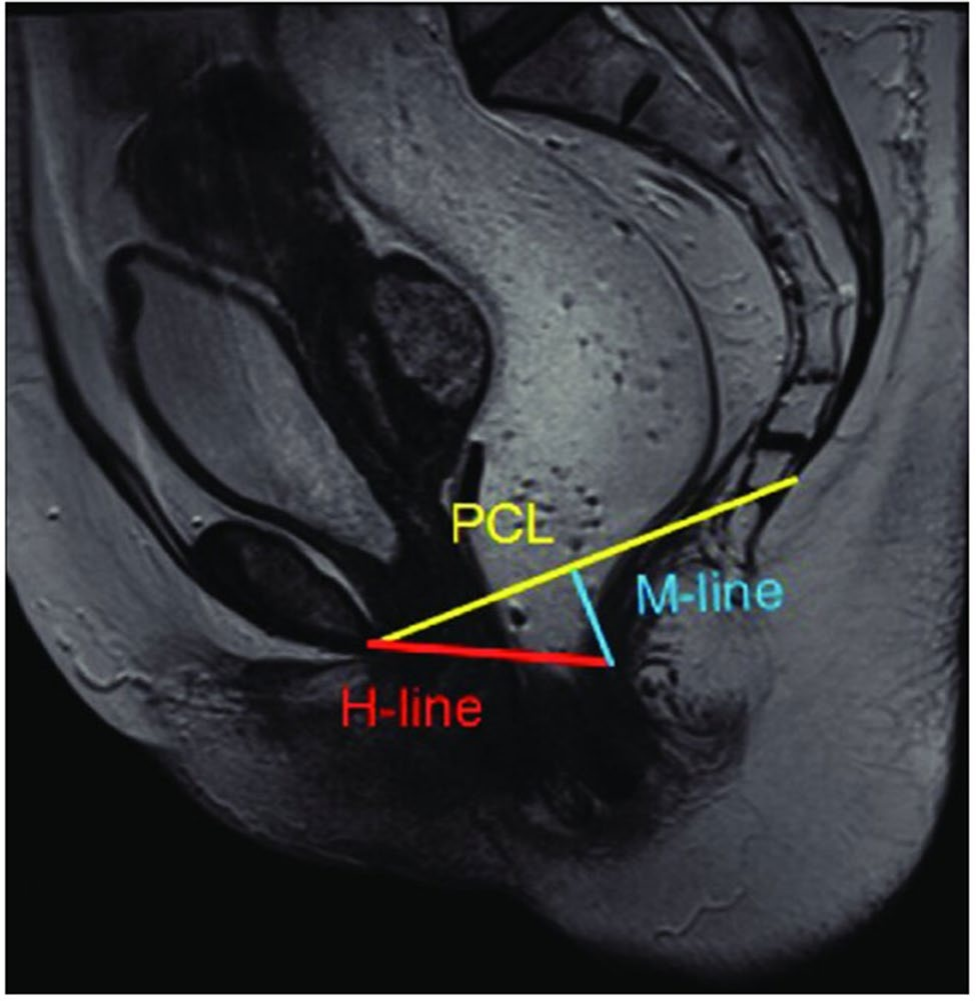
T2W sagittal section of the pelvic magnetic resonance imaging depicting the normal pubococcygeal line (PCL) (yellow), H line (red) and the M line (blue). PCL is between the inferior margin of symphysis pubis and the tip of the coccyx. H-line is the distance between the inferior border of the pubic symphysis and the posterior wall of the rectum at the level of the anorectal junction and indicates the width of the levator hiatus. M-line is the vertical line drawn perpendicularly from the PCL to the posterior end of the H line. M line indicates the degree of decent of the levator hiatus or the degree of pelvic floor laxity

Regarding levatoplasty, all procedures were performed under general anesthesia with the patient in Lithotomy position and the surgeon positioned between the legs of the patient. The patients were firmly secured to the operating table to allow for placement in the anti- Trendelenburg position as required. U- curved incision posterior to the anal verge, dissection was done till Levator ani reached, approximation of both limbs (puborectalis) with polydioxanone sutures (PDS) 3 − 0. (Figures 2, 3 and 4). This was a posterior levatorplasty which we think is more anatomical. All operations (levatorplasty) were done by the same operator.

### Follow up

All patients were assessed post-operatively for the following: Early and late postoperative complications, effect of the procedure on Wexner score, effect of the procedure on recurrence rate, if recurrence occurred, we do MRI defecography, effect of the procedure on postoperative pain according to Visual Analogue Scale (VAS), presence of dyspareunia. postoperative hospital stays. After surgery Patients were followed up to 12 months. The follow up was done monthly through the whole period of the 12 months follow up by clinic visits. Any investigations such as MRI defecography was only done to patients who experience recurrence or recurrence of symptoms during any point of follow up.

### Statistical analysis

Analysis of data was done using SPSS (statistical program for social science version 21) as follows: Description of quantitative variables as mean, median, standard deviation (SD) and interquartile range, description of qualitative variables as number and percentage, repeated measure ANOVA test was used to compare quantitative variables, in parametric data (SD < 30% mean). Mann Whitney test was used instead of unpaired t-test in non- parametric data (SD > 30%mean). Related-Samples Friedman’s Two-Way Analysis of Variance by Ranks test was used instead of repeated measure ANOVA in non- parametric data (SD > 30%mean). P-value < 0.05 was considered statistically significant in all aspects of comparison. This was a time-based study, it ends up with 30 patients in the prospective group then we randomly get other 30 patients with rectal prolapse with wide pelvic hiatus from our records, compared both groups (regarding demographic data and clinical presentation) and standardized the operation to ratio 1:1 in both groups to make the data statistically comparable and reduce biases.

### Sample size calculation and study power

The primary outcome was the rate of recurrence, in similar study by Chun et al. published in 2004 titled “Perineal rectosigmoidectomy for rectal prolapse role of levatorplasty” [19], Using MedCalc software version 14 (MedCalc software bvba, Ostend, Belgium), 26 patients in each group will be needed to reach study power of 80%. Adding 10% for possible dropouts makes the sample size to be at least 28–30 patients in each group.

## Results

The reduction of Wexner-Score and recurrence rate were assessed through the whole period of follow up and at the end of the 12-month period of follow up. Any patient who experiences recurrence of symptoms were investigated for recurrence by MRI defecography at any point of follow up.

### 1) Change in Wexner score over time for Incontinence

In all studied patients there was a decrease in Wexner score over time (pre-operative, post-operative) with a statistically significant difference with p value < 0.001 (Table 2).

**Table 2:** Wexner score changes over time in all studied patients

		*n* = 60
Wexner Pre	Median (IQR)	15 (11–17)
	Range	3–22
Wexner Post	Median (IQR)	0 (0–3)
	Range	0–19
**Willcoxon Rank test**		**-6.459**
**P-value**		**< 0.001 (HS)**

Table (3) shows comparison between both groups regarding Wexner score pre and post operative. There was no statistically significant difference between both groups as shown.

**Table 3:** Comparison between both groups regarding wexner score pre-operative and post-operative in all patients

		With Levatorplasty	Without Levatorplasty	Test value‡	P-value	Sig.
		No. = 30	No. = 30			
Wexner Pre	Median (IQR)	15 (12–17)	15 (10–17)	-0.037	0.970	NS
	Range	8–20	3–22			
Wexner Post	Median (IQR)	0 (0–1)	1 (0–7)	-1.779	0.075	NS
	Range	0–16	0–19			
**Willcoxon Rank test**		**-4.710**	**-4.464**			
**P-value**		**< 0.001 (HS)**	**< 0.001 (HS)**			

### 2) Recurrence

Recurrence in all studied patients was 7 patients (11.7%) while 53 patients show no recurrence (88.3%). The Postoperative Recurrence in levatorplasty group was 1 (3.3%) and was 6 patients in the other control group (20%) with statistically significant difference (P value 0.044). **Leveatorplasty clearly decreases the rate of recurrence.**

### 3) Post-operative pain

The mean post-operative pain in all studied patients was (4.30 ± 1.18), Range [2–7]. Pain was assessed according to VAS score: 0 No pain, 1:3 mild pain, 4:6 moderate pain, 7:9 severe pain and10 worst pain. Mean Postoperative pain in levatorplasty group was (4.37 ± 1.27) while mean postoperative pain in without levatorplasty group was (4.23 ± 1.10) with no statistically significant difference (P value 0.433).

### 4) Dyspareunia

Dyspareunia occurred in 9 patients (15%) while 51 patients (85%) show no dyspareunia. Dyspareunia occurred in 5 patients (16.7%) in levatorplasty group, while dyspareunia occurred in 4 patients (13.3%) in the other group with no statistically significant difference (P value 0.131).

### 5) Operative time

Mean operative time in both groups was 44.05 ± 13.75 min with a range (18–90 min). Mean operative time in levatorplasty was (47.33 ± 13.00) while mean operative time in Without levatorplasty group was (40.77 ± 13.90) with no statistically significant difference (P value 0.064).

### 6) Operations in both groups and subgroup analysis

Table (4) shows operative data in both groups. We standardized the operation to ratio 1:1 in both groups to make the data statistically comparable.

**Table 4:** Operative data in both groups

		No.	%
With Levatorplasty	ALTEMEIER’S operation	5	16.7%
	POPS operation	5	16.7%
	Rectopexy operation	10	33.3%
	STARR operation	10	33.3%
Without Levatorplasty	ALTEMEIER’S operation	5	16.7%
	POPS operation	5	16.7%
	Rectopexy operation	10	33.3%
	STARR operation	10	33.3%

### A) Altemeier’s Operation

Five patients from each group were conducted to ALTEMEIER’S procedure. Table (5) Shows no difference between both regarding all aspects of comparison apart from intra-operative time shows statistically significant shorter time in without levatorplasty group. Also, Table (5) shoes comparison of Wexner scores between both groups.

**Table 5:** Comparison between With Levatorplasty and Without Levatorplasty in ALTEMEIER’S operation

ALTEMEIER’S operation		With levatorplasty	Without levatorplasty	Test value‡	*P*-value	Significance
		*n* = 5	*n* = 5			
Wexner Pre	Median (IQR)	12 (10–12)	12 (10–12)	-0.108	0.914	NS*
	Range	8–13	8–15			
Wexner Post	Median (IQR)	0 (0–1)	1 (0–1)	-0.808	0.419	NS
	Range	0–1	0–10			
**Willcoxon Rank test**		**-2.032**	**-2.032**			
**P-value**		**0.042 (S)**	**0.042 (S)**			
**Mean post-operative pain**		4.2 ± 0.84	4.2 ± 0.84		1	NS
**Dyspareunia**		0	1		0.292	NS
**Recurrence**		0	1		0.292	NS
**Mean operative time**		36.00 ± 4.18	24.40 ± 4.04		0.002	S**

* Statistically non-significant

** Statistically Significant

### B) POPS operation

Five patients from each group were conducted to POPS procedure. Table (6) Shows no difference between both groups regarding all aspects of comparison apart from intra-operative time shows statistically significant shorter time in without levatorplasty group. Also, Table (6) shoes comparison of Wexner scores between both groups.

**Table 6:** Comparison between With Levatorplasty and Without Levatorplasty in POPS operation

POPS operation		With levatorplasty	Without levatorplasty	Test value‡	*P*-value	Significance
		*n* = 5	*n* = 5			
Wexner Pre	Median (IQR)	17 (16–18)	19 (15–20)	-0.629	0.530	NS*
	Range	12–20	13–22			
Wexner Post	Median (IQR)	0 (0–3)	3 (0–4)	-0.671	0.502	NS
	Range	0–10	0–15			
**Willcoxon Rank test**		**-2.023**	**-1.841**			
**P-value**		**0.043 (S)**	**0.066 (NS)**			
**Mean post-operative pain**		4.4 ± 1.52	4.4 ± 1.14		1	NS
**Dyspareunia**		1	1		1	NS
**Recurrence**		0	1		0.292	NS
**Mean operative time**		31.00 ± 2.24	20.20 ± 1.92		0.0001	S**

### C) ventral mesh rectopexy operation

Ten patients from each group were conducted to Ventral Mesh Rectopexy procedure. Table (7) Shows no difference between both regarding all aspects of comparison. Also, Table (7) shows shoes comparison of Wexner scores between both groups.

**Table 7:** Comparison between With Levatorplasty and Without Levatorplasty in Ventral Mesh Rectopexy operation

Rectopexy operation		With levatorplasty	Without levatorplasty	Test value‡	*P*-value	Sig.
		No. = 10	No. = 10			
Wexner Pre	Median (IQR)	16 (14–18)	16 (8–17)	-0.499	0.618	NS
	Range	8–20	3–20			
Wexner Post	Median (IQR)	0 (0–3)	3 (0–8)	-1.096	0.273	NS
	Range	0–16	0–19			
**Willcoxon Rank test**		**-2.673**	**-2.375**			
**P-value**		**0.008 (HS)**	**0.018 (S)**			
**Mean post-operative pain**		4.2 ± 1.55	4.3 ± 1.49		0.8	NS
**Dyspareunia**		2	1		0.5	NS
**Recurrence**		1	2		0.5	NS
**Mean operative time**		58. ± 12.29	52.3 ± 5.1		0.19	NS

### D) STARR operation

Ten patients from each group were conducted to STARR procedure. Table (8) Shows no difference between both regarding all aspects of comparison. Also, Table (8) shoes comparison of Wexner scores between both groups.

**Table 8:** Comparison between With Levatorplasty and Without Levatorplasty in STARR operation

STARR operation		With levatorplasty	Without levatorplasty	Test value‡	*P*-value	Sig.
		*n* = 10	*n* = 10			
Wexner Pre	Median (IQR)	13 (10–17)	15 (10–17)	-0.266	0.790	NS
	Range	8–20	8–20			
Wexner Post	Median (IQR)	0 (0–1)	0.5 (0–1)	0.991	0.322	NS
	Range	0–3	0–10			
**Willcoxon Rank test**		**-2.805**	**-2.805**			
**P-value**		**0.005 (HS)**	**0.005 (HS)**			
**Mean post-operative pain**		4.6 ± 1.17	4.1 ± 0.88)		0.295	NS
**Dyspareunia**		2	1		0.5	NS
**Recurrence**		0	2		0.136	NS
**Mean operative time**		50.5 ± 5.1	47.7 ± 1.64		0.1	NS

## Discussion

Complete rectal prolapse is defined as complete protrusion of all layers of the rectum from the anal verge. Although many theories have been suggested for its development, but the most widely accepted is the weakness and laxity of the connective tissue attachments of the rectal mucosa. Other risk factors include multiparity, traumatic perineal injury, obesity as well as psychiatric and connective tissue diseases [11].

Rectal prolapse is a debilitating condition that can cause symptoms of fecal incontinence, obstructed defecation, incomplete evacuation of the rectum, sensation of rectal pressure, and pain. Although there are several conservative measures, surgical management remains the mainstay option which aims to improving bowel function and quality of life. Recently, several combined surgical procedures have been evolved as part of a multidisciplinary evaluation and treatment that can be offered to the patients. The American College of Surgeons National Surgical Quality of Improvement Program (ACS NSQIP) study has recently demonstrated that the number of combined rectal prolapse and pelvic organ prolapse surgeries has increased from 2.6 to 7% over the last decade [12–14].

The main aim of treatment is the correction of the prolapse and to treat associated functional symptoms such as incontinence and/or constipation. This goal can be achieved by several procedures, either by fixation of the rectum to the sacrum, resection, plication of the redundant bowel or combining several procedures together. There are different approaches which can be done either trans anal/perineal or transabdominal. Recently, there has been increasing evidence that prolapse development is strongly associated with levator hiatus size. Multiple surgical studies have suggested that an enlarged pelvic hiatus may be associated with prolapse recurrence [15–17]. 

The iliococcygeus muscle is a thin, fan-shaped structure that takes curved shape inferiorly when observed in the coronal plane. It is inserted laterally to the pelvic sidewalls. Posteriorly it forms a raphe that blends with the attachment of the external anal sphincter to form the anococcygeal ligament which is inserted on the coccyx. The puborectalis muscle is a U-shaped sling inserted on the inner pubis as it passes around the anorectal junction. The levator plate angle plays an important role in normal support, normal angle (4.5 ± 0.7). Accordingly, levator ani defects and associated fascial defects further increase the risk of pelvic floor dysfunction [18]. 

Our results as discussed above showed that levatoplasty has an added value when it is performed with the classical repair of rectal prolapse – in patients with wide pelvic hiatus- mainly in decreasing recurrence rate. Also, it shows numerical improvement in the control of symptoms (Wexner’s score) although statistically insignificant which may be due to the small number of patients allocated to each surgery. Also, regarding other aspects of comparison such as post-operative, dyspareunia and mean operative time, there was no statistically significant difference between both groups. Interestingly, performing levatorplasty with other rectal prolapse surgery did not add significant pain.

In a study by *Chun et al. published in 2004*, a total of 109 consecutive patients (10 men) underwent 120 perineal procedures: perineal rectosigmoidectomy (PRS) or perineal rectosigmoidectomy with levatorplasty (PRSL). Regarding change of Wexner score over time Both groups had significant improvements in postoperative incontinence score (*p* < 0.0001) [19]. Th is going with the results of our study where there was a decrease in Wexner score over time with a statistically significant difference with p value < 0.001. Regarding Recurrence rates and mean time interval to recurrence in *Chun et al.* ‘s study was, respectively, 20.6% and 45.5 months in PRS compared to 7.7% and 13.3 months in PRSL (p = 0.049). Also, in our study Postoperative Recurrence in Levatorplasty group was 1 (3.3%) was less than that in without Levatorplasty group which was 6 (20%) with a statistically significant difference (P value = 0.044) [19].

Regarding their operative time, mean duration of surgery was 78.1 min (SD = 25.9) and 97.6 min (SD = 32.3) in PRS and PRSL, respectively (*p* = 0.002) [19]. However, in our study mean operative time in levatorplasty group (47.33 ± 13.00), while mean operative time in without levatorplasty group (40.77 ± 13.90) with no statistically significant difference (P value = 0.433) [19]. 

In a study by J Randall et al. published in colorectal disease in 2016 discussing Laparoscopic ventral mesh rectopexy in 120 patients presenting with rectal prolapse shows recurrence rate of 3% with good control of symptoms [20]. Regarding perineal procedures, a study discussing Stappled resection [21] and another one discussing perineal recto-sigmoidectomy [22] shows high rate of recurrence and only fair control of symptoms but both procedures are fast and safe procedures which are suitable for old frail patients. However, all those studies did not mention the size of Levator hiatus and its effect like most of the literature available. Therefore, we think that our study will add to literature regarding the role of Levatorplasty in rectal prolapse surgery.

### Conclusion and recommendations

Rectal prolapse repair combined with levatorplasty in patients complaining of rectal prolapse with wide pelvic hiatus have better outcome in decreasing recurrence rate. Also, it shows better control of clinical symptoms although statistically insignificant. Hence, we recommend rectal prolapse repair to be combined with levatorplasty in patients complaining of rectal prolapse with wide pelvic hiatus, however larger studies on larger group pf patients are needed. Also, proper detailed assessment and tailored treatment plan for each patient are the key for better outcome.

### Strengths and limitations

#### Limitations

Include sample size and follow-up duration, so we recommend more studies to be done with bigger sample size and long follow-up duration. However, strengths of our study include a relatively good number of patients that was adjusted to 1:1 ratio and were comparable regarding demographic data and co-morbidities. Also, investigating the role of wide pelvic hiatus and the role of its repair which we think is a forgotten issue in the management of rectal prolapse.

## Data Availability

Availability of data: The datasets used and/or analyzed during the current study are available from the corresponding author on reasonable request.

## Declarations

### Abbreviations

all abbreviations are mentioned in detail the first time they appear in the text.
